# Multispectral optoacoustic tomography of salivary glands in patients with clinically suspected Sjögren’s disease: A pilot study

**DOI:** 10.1016/j.pacs.2025.100778

**Published:** 2025-11-01

**Authors:** Rik de Jong, Milou E. Noltes, Hendrika Bootsma, Gooitzen M. van Dam, Andor W.J.M. Glaudemans, Schelto Kruijff, Riemer H.J.A. Slart, Alja Stel, Max J.H. Witjes, Konstantina Delli, Jasper Vonk

**Affiliations:** a Department of Nuclear Medicine and Molecular Imaging, University Medical Center Groningen, University of Groningen, Groningen, the Netherlands; b Department of Surgery, University of Groningen, University Medical Center Groningen, Groningen, the Netherlands; c Department of Rheumatology and Clinical Immunology, University Medical Center Groningen, University of Groningen, Groningen, the Netherlands; d AxelaRx/TRACER B.V., Groningen, the Netherlands; e Department of Biomedical Photonic Imaging, Faculty of Science and Technology, University of Twente, Enschede, the Netherlands; f Department of Oral and Maxillofacial Surgery, University Medical Center Groningen, University of Groningen, Groningen, the Netherlands; g Department of Radiology, University Medical Center Groningen, University of Groningen, Groningen, the Netherlands

**Keywords:** Multispectral optoacoustic tomography, Photoacoustic imaging, Sjögren’s disease, ACR-EULAR, Salivary glands, Ultrasound

## Abstract

Sjögren’s disease (SjD) is a systemic auto-immune disease characterized by salivary gland inflammation and glandular dysfunction. Diagnosis is challenging due to its heterogeneity and currently relies on a variety of tests that are present in the ACR-EULAR classification criteria. These include invasive and resource-intensive tests, highlighting the unmet need for a single, accurate, non-invasive diagnostic modality. Multispectral optoacoustic tomography (MSOT), enabling functional imaging of hemoglobin-related parameters, may address this gap. This pilot study evaluates MSOT's potential for salivary gland imaging in patients suspected of SjD. This study included 20 patients clinically suspected of SjD. Which underwent MSOT imaging of the major salivary glands, alongside the full ACR-EULAR diagnostic workup, including serology, salivary gland biopsy, and sialometry, alongside salivary gland ultrasound. MSOT parameters were compared to standard of care diagnostic modalities and ultrasound scoring systems. A novel MSOT scoring system based on 800 nm hemoglobin signals was developed to evaluate diagnostic performance. Patients classified as SjD (n = 13) show significantly higher hemoglobin-related signals compared to non-SjD patients (n = 7). When ≥ 2 salivary glands, either submandibular or parotid, exceeded their predefined MSOT cut-off values of 371.6 a.u. and 374.2 a.u., respectively, MSOT achieved 92 % sensitivity and 100 % specificity for SjD classification, outperforming other diagnostic tests and established ultrasound scoring systems. MSOT shows promise as non-invasive imaging modality for SjD classification, and may offer higher sensitivity compared to established ultrasound scoring systems and other diagnostic tests. These explorative findings support further investigation of MSOT as non-invasive diagnostic tool in SjD.

## 1 Introduction

Sjögren’s disease (SjD) is a systemic auto-immune disease characterized by chronic inflammation of the exocrine glands, primarily affecting the lacrimal and salivary glands [1]. Its prevalence is estimated at 61 cases per 100,000 inhabitants, with a typical onset in middle-aged adults, although it can manifest at any age. SjD may exist independently or alongside other autoimmune diseases, like rheumatoid arthritis or systemic lupus erythematosus [2]. Clinically, SjD patients often present with hallmark symptoms such as dry eyes and mouth [3]. However, due to the systemic nature of the disease, patients may also experience additional symptoms such as fatigue and arthritis, contributing to its diagnostic complexity [4].

Accurate and timely diagnosis of SjD remains a significant challenge due to its clinical heterogeneity and overlapping symptoms with other diseases [5], [6]. No single diagnostic test offers both high sensitivity and specificity. Instead, diagnosis relies on a variety of diagnostic tests and is still based on expert opinion [7], [8]. Numerous classification criteria for SjD have been proposed, with the 2016 American College of Rheumatology/European League Against Rheumatism (ACR-EULAR) classification criteria being the most widely accepted standard [9]. These criteria were originally designed for patient recruitment in clinical trials to ensure homogeneous research populations but are also often used alongside expert opinion for clinical diagnostic purposes. The criteria require a multifaceted and often invasive diagnostic workup, involving salivary gland biopsy, the presence of anti-SSA/Ro antibodies, unstimulated whole saliva flow (UWS), Schirmer’s test and Ocular Staining Score (OSS). While the ACR-EULAR classification criteria achieve high sensitivity and specificity, they are resource-intensive and may not always provide a timely diagnosis [10]. There is an unmet clinical need for a fast, reliable, simple and non-invasive diagnostic test for SjD, with a high sensitivity and specificity.

Salivary gland ultrasound (SGUS) has emerged as a valuable tool for evaluating the salivary glands, aiding in non-invasive SjD diagnosis, longitudinal monitoring of the salivary glands and monitoring treatment response [11], [12], [13]. As SGUS has shown high predictive value for identifying SjD, there have been recommendations to incorporate SGUS into the ACR-EULAR classification criteria [14]. However, SGUS relies on the detection of structural changes, which may be subtle or absent in early stages of the disease. Secondly, it is unclear whether SGUS can assess subtle differences in salivary gland involvement associated with disease progression.

To overcome these limitations, molecular imaging approaches may detect SjD by identifying subtle biochemical changes that could precede structural changes in disease development. Multispectral optoacoustic tomography (MSOT) is a non-invasive hybrid imaging modality that complements ultrasound (US) by providing functional and molecular information [15]. MSOT is based on the optoacoustic effect, whereby light absorption by tissue chromophores triggers thermoelastic expansion, generating US waves detectable by a transducer array. This enables the visualization of rich optical contrast with ultrasonic spatial resolution [16]. MSOT is able to differentiate between chromophores such as deoxyhemoglobin (HbR), oxyhemoglobin (HbO_2_), melanin and lipid, through their unique absorption spectra [17]. This allows MSOT to offer detailed insights into biochemical processes, making it a valuable non-invasive tool for cancer detection, and accurate diagnosis and evaluation of inflammatory processes [18], [19], [20], [21], [22].

In the context of SjD, MSOT might be an asset, due to its ability to quantify hemoglobin concentration and distribution to assess microvascular changes, related to hyperemia and hypervascularization. MSOT has been successfully used in autoimmune diseases such as rheumatoid arthritis and Crohn’s disease to assess microvascular changes, serving as biomarkers for inflammatory activity [23], [24], [25]. Salivary gland inflammation in SjD patients is also related to hyperemia and hypervascularization, resulting from vascular remodeling in the inflamed tissue [26]. Earlier Color and Power Doppler studies have shown increased blood flow towards the salivary glands of SjD patients [27]. However, Doppler imaging is not suited to visualize slow-flowing blood in the microcirculation, whereas these microvascular alterations could be a relevant biomarker for SjD. Previous studies have shown that MSOT is more effective than Doppler imaging for visualizing microvascular structures [28]. This suggests that MSOT could provide a more sensitive and objective approach for detecting microvascular changes in SjD, potentially improving diagnostic value and disease monitoring.

In this pilot study, we aimed to evaluate the potential of MSOT as a diagnostic tool for salivary gland assessment in patients with clinical suspicion of SjD. Hemoglobin-related MSOT signals were compared with standard-of-care diagnostic tests, including SGUS, parotid gland biopsies, sialometry, and serological markers, as well as the ACR-EULAR classification criteria.

## 2 Materials and methods

### 2.1 Study design and patients

This single-center, pilot study was performed at the University Medical Center Groningen (UMCG), a tertiary referral and SjD expertise center. Patients ≥ 18 years clinically suspected of SjD were eligible for inclusion. Patients included in this study were part of the RESULT (REgistry of Sjögren syndrome in UMCG – LongiTudinal) cohort [29]. The study protocol was approved by the Medical Ethics Committee of the University Medical Center Groningen (METc 2019/633). The study was performed in accordance with the Helsinki Declaration (adapted version 2013, Fortaleza, Brazil). Written informed consent was obtained from all participants included in the study prior to any study-related procedure. The study was registered at clinicaltrials.gov (NCT06740175).

### 2.2 Diagnostic workup/ACR-EULAR classification criteria

All patients underwent clinical diagnostic evaluation, including all diagnostic tests of the 2016 ACR-EULAR classification criteria, comprising parotid gland biopsy, anti SSA/Ro antibody testing, ocular staining score (OSS), Schirmer’s test, and unstimulated whole saliva flow (UWS) [9]. Within the UMCG a parotid gland biopsies is performed instead of a labial gland biopsy because of comparable diagnostic accuracy and less morbidity [30]. Patients with a combined ACR-EULAR score ≥ 4 were classified as SjD [9]. In addition, all patients underwent ultrasound imaging of the major salivary glands according to standard of care in our center.

### 2.3 Ultrasound evaluation

SGUS of the major salivary glands was performed according to standard of care at the UMCG. US was performed by trained operators using a clinical US device (Esaote MyLab Twice, Easaote SpA, Genoa, Italy) equipped with a high-resolution linear probe (3–13 MHz). Patients were imaged in supine position with their neck slightly extended and rotated away from the examined side. The Hocevar scoring system was used as described previously (Hocevar-score 0–48) [31]. A Hocevar score ≥ 15 was considered positive (i.e. abnormal) [12]. The Hocevar scores were also converted to the recently described OMERACT-score, where an OMERACT score ≥ 2 in at least one salivary gland is considered abnormal [32].

### 2.4 MSOT setup and imaging procedures

The MSOT procedures were performed using a clinical hybrid US-MSOT system (MSOT Acuity Echo prototype; iThera Medical GmbH, Germany), as previously described in detail [33]. Briefly, this system detects both US and optoacoustic signal. A 25 Hz pulsed Nd:YAG laser is used to illuminate the tissue of interest with a maximum pulse energy of 30 mJ, in accordance with the American National Standards Institute safety limit for laser exposure [34]. The handheld concave detector array (256 transducer elements with a center frequency of 3.4 MHz and bandwidth of 60 % in receive-transmit mode, 125° angular coverage) provides optoacoustic (OA) resolution of ∼250 nm with a field of view of 40 × 40 mm.

MSOT procedures were performed directly before parotid biopsy procedures, to ensure that the observed signals were attributed solely to the disease and were not affected by the biopsy. To minimize variability in salivary gland activity, all patients were required to fast (i.e. both food and drink) for a minimum of one hour prior to the MSOT procedure. As with standard of care US, patients were imaged with MSOT in supine position, with their neck in slight hyperextension and their head rotated away from the examined side. Both parotid and submandibular glands were imaged bilaterally. Imaging was performed in a dedicated MSOT-imaging room following all safety regulations for use of class IV lasers (e.g. laser interlock system, laser safety goggles).

MSOT imaging was acquired at 14 distinct wavelengths ranging from 680 to 1195 nm (specifically; 680, 700, 730, 760, 800, 850, 900, 930, 970, 1000, 1030, 1064, 1100, 1195 nm). The selected wavelengths facilitate the detection and reliable spectral unmixing of tissue chromophores. For each gland, at least three scans were performed, resulting in a minimum of 12 scans per patient, with additional scans conducted as necessary.

### 2.5 Data processing, image reconstruction and data analysis

At least 12 scans were available per patient, comprising three scans per gland (i.e. two parotid and two submandibular glands). In the data processing stage, all three scans were utilized. If more than three scans were available, the three scans with the least reflection artifacts and movement (as measured by the iThera MSOT Acuity Echo), and the highest quality, as determined by expert assessment, were selected. Each scan consists of multiple frame stacks that represent different time intervals. For each scan, a total of three frames where evaluated and averaged, to increase the signal-to-noise ratio. The final data point per gland is the average value of all three scans.

To quantify the MSOT signal, a standardized elliptical region of interest (ROI) (area: 60,49 mm^2^, height 5,4 mm, width 14,0 mm) was drawn on the selected frames to maintain consistency throughout the data processing. This approach mitigates the effects of signal reduction at the edges and depth of the field of view (FOV) [35]. Given that light absorption is most pronounced in the center of the FOV, a ROI drawn around the entire parotid gland would result in diminished signal differences [36]. An elliptical ROI was drawn at the surface of the salivary gland of interest on the B-mode US image. ROI placement and data analysis was performed by operators blinded to the patients final diagnosis or classification status.

Both single wavelengths (SWLs) (700, 730, 760, 800 and 850 nm) and unmixing spectra, including HbR, HbO_2_, and total hemoglobin (HbT), were computed. SWLs constitute to the absorption of all molecules within the sample at this specific wavelength, while spectral unmixing allows for specific molecular quantification. During this study, a back-projection algorithm was used for image reconstruction, and linear unmixing results of five SWLs (specifically; 700, 730, 760, 800 and 850 nm) were used to compute the unmixing spectra of HbR and HbO_2_. HbT represents the sum of HbR and HbO_2_ and is also represented at the isosbestic point of hemoglobin which is 800 nm [37]. The results are expressed as arbitrary units (a.u.). For the reliability of the data, a threshold is implemented into the algorithm to exclude negative pixels from the spectral unmixing process. For each pixel location across the range of wavelengths, some may yield negative intensity values, while others are positive. If the number of wavelengths exhibiting negative values at a specific pixel surpasses the predetermined threshold, that pixel is excluded from the spectral unmixing analysis, subsequently being assigned a ‘Not a Number’ (NaN) value. Imaging data processing was conducted using the software provided by the manufacturer (iLabs V 1.3.32, iThera Medical GmbH, Munich, Germany).

### 2.6 ROI depth difference between parotid and submandibular glands

The intensity of the optoacoustic signal is proportional to the light energy deposition, which is essentially the product of the absorption coefficient and the local light fluence. As light fluence decreases with imaging depth, MSOT signal is reduced with increasing depths [38]. Therefore, the data from the parotid glands and submandibular glands were analyzed separately to avoid depth-effects biasing our analysis.

### 2.7 Statistical analysis

Descriptive statistics were performed on the patient demographics and clinical data. Due to the limited sample size of this pilot study, all data was considered non-normally distributed. Data were presented as median and interquartile range (IQR), frequencies, and percentages. MSOT data were collected as means in case of SWLs, and unmixing spectra obtained with linear spectral unmixing, and are visualized as box and whiskers plots, with minimum and maximum, and 25 % upper and lower quartile of the median.

The Mann-Whitney *U* test was used to analyze distributions of single wavelength signals and unmixing spectra of HbR, HbO_2_ and HbT in subjects classified as SjD and non-SjD. An exploratory Receiver Operating Characteristic (ROC) curve analysis was conducted to evaluate the diagnostic performance of the different single wavelengths and unmixing spectra in differentiating between SjD and non-SjD. The optimal cut-off values for both parameters were determined using Youden’s index, describing the cut-off value in which the highest combination of sensitivity and specificity is found. A value above the cut-off was considered indicative of SjD. The area under the ROC curve (AUC) was reported to quantify the predictive ability of the parameters in differentiating between the two groups (no discrimination 0–0.5; poor accuracy 0.5–0.7; fair 0.7–0.8; good 0.8–0.9; excellent 0.9–1.0) [39]. All imaging scoring systems and other diagnostic tests were compared to the ACR-EULAR classification criteria. All statistical analyses were performed using SPSS software, version 23, and GraphPad Prism 9.90 (GraphPad Software Inc.).

## 3 Results

### 3.1 Patient demographics

Between March and October 2021, 20 consecutive female patients with clinical suspicion of SjD were included in the study, with a median age of 57 (26 – 70) years. All patients underwent parotid gland biopsy per standard of care. One patient did not complete the OSS, Schirmer test, and US examination. Based on the ACR-EULAR classification criteria, 13 patients were classified as SjD and 7 as non-SjD. One patient with SjD classification was also coincidentally diagnosed with mucosa-associated lymphoid tissue (MALT) lymphoma.

MSOT imaging was successfully completed in all patients within 15 min, without any adverse events. Nineteen patients had all four glands imaged using MSOT. In one patient, only three glands were imaged due to a prior surgical removal of a submandibular gland. In total, the MSOT data set comprised images of 26 parotid and 25 submandibular glands in the SjD group, and 14 parotid and submandibular glands in the non-SjD group, with each gland representing one data point. Patient demographics are summarized in Table 1.

**Table 1:** Patient demographics.

Characteristics	Total group (n = 20; 100 %)	SjD (n = 13; 65 %)	Non-SjD (n = 7; 35 %)
Female	20 (100 %)	13 (100 %)	7 (100 %)
Age (years), median (range)	57 (26 – 70)	57 (26 – 70)	49 (39 – 64)
Parotid gland biopsy, focus score ≥ 1	14 (70 %)	12 (92 %)	2 (29 %)
Anti-SSA antibodies	9 (45 %)	9 (69 %)	0 (0 %)
UWS ≤ 0.1 mL/min	12 (60 %)	9 (69 %)	3 (43 %)
OSS ≥ 5	1 (5 %)	0 (0 %)	1 (14 %)
Schirmer’s test ≤ 5 mm/5 min	11 (55 %)	8 (62 %)	3 (43 %)
SGUS Hocevar score ≥ 15	4 (20 %)	4 (31 %)	0 (0 %)
SGUS OMERACT score ≥ 2	5 (25 %)	4 (31 %)	1 (14 %)

UWS (unstimulated whole saliva flow), OSS (ocular staining score), SjD (Sjogren’s disease). SGUS (salivary gland ultrasound)

### 3.2 Increased hemoglobin-related signals in parotid and submandibular glands of SjD patients

**Fig. 1:**
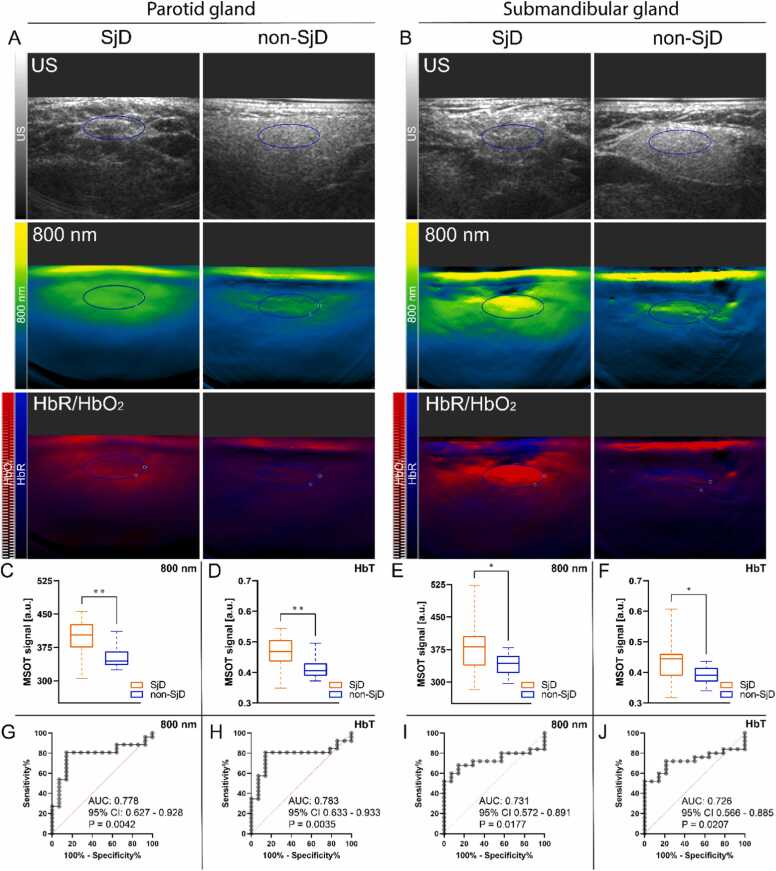
**MSOT of hemoglobin parameters enables discrimination between SjD and non-SjD patients. A,B.** Representative US, 800 nm, and spectrally unmixed HbR /HbO_2_ images of the parotid gland and submandibular gland, respectively of SjD patients non-SjD patients. **C-F.** Hemoglobin-based signals are significantly increased in SjD patients compared to non-SjD patients, both for all single wavelengths and spectrally unmixed hemoglobin parameters. mean coefficients obtained with spectral unmixing. The SWL and spectrally unmixed parameter with highest diagnostic accuracy are highlighted; 800 nm and HbT. **G-J.** Receiver operator characteristics curves of 800 nm and HbT, MSOT readouts show fair performance for both parotid glands and submandibular glands. US (Ultrasound), HbR (deoxyhemoglobin), HbO_2_ (oxyhemoglobin), SjD (Sjögren’s disease), SWL (single wavelength), MSOT (multispectral optoacoustic imaging).

Quantitative MSOT analysis showed increased signal in SjD patients across all SWLs (i.e. 700, 730, 760, 800, 850 nm) and unmixing spectra (i.e. HbR, HbO_2_, HbT), as described in Supplementary table S1 (Supplementary Material). Representative US and MSOT images of the parotid and the submandibular glands are shown in Fig 1B and A, highlighting visual differences in signal intensity between the SjD and non-SjD group for both 800 nm single wavelength (SWL) and HbR/HbO_2_ images.

The most pronounced difference in SWL measurements was observed at 800 nm, corresponding to the isosbestic point of hemoglobin, in the parotid glands [37]. Patients with SjD showed significantly higher median signal of 403.1 (374.6 – 428.0 a.u.), compared to 344.9 (335.4 – 366.6 a.u.) in the non-SjD group (p = 0.0035), as shown in Fig. 1C,E.

Among the obtained unmixing spectra measurements, HbT in the parotid gland showed the most significant difference between groups, shown in Fig. 1D and Supplementary table S1 (Supplementary Material). The SjD group showed a median signal of 0.47 (0.44 – 0.51 a.u.), compared to 0.41 (0.39 –0.43 a.u.) in the non-SjD group (p = 0.0028). In the submandibular gland, HbT was the only unmixing spectrum that exhibited a significant difference, as shown in Fig. 1F.

Patients with SjD are at risk of developing MALT lymphoma, a malignant tumor that can affect the salivary glands [40]. One patient coincidentally diagnosed with MALT lymphoma in the right parotid gland was imaged using MSOT and is presented in Supplementary Fig. S1 (Supplementary Material). SWL measurements showed slightly higher median 800 nm signal in the right parotid gland (800 nm; 438.1 [424.6 – 454.1 a.u.]) compared to the left parotid gland (800 nm signal; 410.6 [397.3 – 419.4 a.u.]). This small difference is not evident in the MSOT images (Supplementary Material, Supplementary Fig. S1).

### 3.3 Diagnostic accuracy of MSOT per salivary gland to classify SjD patients according to the ACR-EULAR classification criteria

To evaluate the diagnostic potential of MSOT to differentiate SjD from non-SjD patients, explorative ROC analyses were performed on all SWLs and unmixing spectra for both parotid and submandibular glands, using the final ACR-EULAR classification outcome as reference standard.

In the parotid gland (Fig. 1G,H), SWL measurements at 800 nm achieved an AUC of 0.778 (95 % CI 0.627 – 0.928; Fig. 1G), comprising an optimal cut-off value of 371.6 a.u. as determined by Youden’s index. This yielded 80.8 % sensitivity, 85.7 % specificity, 70.6 % NPV, 91.3 % PPV, and 82.5 % accuracy. Similarly, HbT in the parotid gland showed an AUC 0.783 (95 % CI 0.633–0.933; Fig. 1H), with 0.43 a.u. as optimal cut-off value, resulting in 80.8 % sensitivity, 85.7 % specificity, 70.6 % NPV, 91.3 % PPV, and 82.5 % accuracy.

In the submandibular gland, 800 nm SWL measurements resulted in an AUC of 0.731 (95 % CI 0.572 – 0.891; Fig. 1I). The cut-off value of 374.2 a.u. as determined by Youden’s index resulted in 60.0 % sensitivity, 92.9 % specificity, 55.6 % NPV, 94.0 % PPV, and 71.8 % accuracy. For HbT, the AUC was 0.726 (95 % CI 0.566 – 0.885; Fig. 1J), with a cut-off value of 0.41 a.u., resulting in 72.0 % sensitivity, 78.6 % specificity, 60.2 % NPV, 86.2 % PPV, and 74.4 % accuracy. The diagnostic performance of both glands are presented in Supplementary table S2 (Supplementary Material).

### 3.4 Development of an MSOT scoring system for SjD classification

Given that the 800 nm SWL analysis consistently showed the highest diagnostic accuracy and is independent of spectral unmixing accuracy, resulting in more reliable data, this parameter was considered for further development of a MSOT scoring system [37]. Based on the previously determined cut-off values for both parotid and submandibular glands, we evaluated the number of glands exceeding the cut-off value, ranging from 0 to 4 glands. Using this approach, a threshold of ≥ 2 affected glands yielded the best classification performance, resulting in 92.3 % sensitivity, 100 % specificity, 87.5 % NPV, 100 % PPV, 87.5 %, and 95 % accuracy (Supplementary Material, Supplementary table S3).

### 3.5 MSOT compared to SGUS scoring systems and diagnostic tests

The diagnostic performance of the MSOT scoring system was compared to the OMERACT and Hocevar SGUS scoring systems, using the ACR-EULAR classification as reference standard. MSOT showed highest diagnostic accuracy (AUC 0.984, 95 % CI 0.938–1.000) compared to OMERACT (AUC 0.582, 95 % CI 0.323 – 0.843) and Hocevar (AUC 0.654, 95 % CI 0.413 – 0.894) as presented in Table 2.

**Table 2:** MSOT, OMERACT, Hocevar, and the diagnostic tests of the ACR-EULAR criteria versus the ACR-EULAR classification outcome as reference standard. UWS (unstimulated whole saliva flow), OSS (ocular staining score).

	Sensitivity (%)	Specificity (%)	NPV (%)	PPV (%)	AUC	Accuracy (%)
Imaging modality vs ACR-EULAR criteria						
MSOT	92.3	100.0	87.5	100.0	0.984	95.0 (18/20)
OMERACT	30.8	85.7	40.0	80.0	0.582	50.0 (10/20)
HOCEVAR	30.8	100.0	43.7	100.0	0.654	55.0(11/20)
Diagnostic tests vs ACR-EULAR classification criteria						
Parotid biopsy	84.6	71.4	71.4	84.6	0.780	80.0 (16/20)
Anti-SSA/Ro	69.2	100.0	63.6	100.0	0.846	80.0 (16/20)
UWS	69.2	57.1	50.0	75.0	0.632	65.0 (13/20)
OSS	14.3	100.0	38.6	100.0	0.571	30.0 (6/20)
Schirmer	61.5	57.1	44.4	72.7	0.593	60.0 (12/20)

Specifically, MSOT demonstrated substantially higher sensitivity (92.3 %) compared to both Hocevar (30.8 %) and OMERACT (30.8 %), while the specificity was comparable, 100.0 % for MSOT and Hocevar, versus 85.7 % for OMERACT. Additional comparisons of all diagnostic tests with the ACR-EULAR classification criteria are also presented in Table 2. A separate analysis comparing SGUS scoring systems and other diagnostic tests to MSOT as a reference standard, is provided in Supplementary table S4 (Supplementary Material).

## 4 Discussion

This pilot study highlights the potential of MSOT as a non-invasive imaging modality for salivary gland evaluation in patients clinically suspected of having SjD. Our findings demonstrate that patients classified as having SjD exhibit significantly increased hemoglobin-related MSOT signals across all SWLs and unmixing spectra (HbR, HbO_2_ and HbT), likely reflecting inflammation-associated hyperemia and microvascular remodeling.

Among individual salivary glands, the highest diagnostic performance was observed in the parotid glands, with both 800 nm SWL and HbT signals resulting in 80 % sensitivity and 85 % specificity. Although submandibular glands showed lower sensitivity (60 % for 800 nm SWL and 72 % for HbT) they maintained similar specificity of 93 % and 79 %, respectively. However, this approach only considers the assessment of individual glands, whereas existing SGUS scoring systems (e.g., Hocevar and OMERACT) include all glands within a unified score [31], [32]. To address this, we developed an MSOT scoring approach that evaluates all glands and classifies patients as positive when ≥ 2 salivary glands exceed their predefined cut-off values. This scoring system achieved 92 % sensitivity and a 100 % specificity, outperforming other diagnostic tests incorporated in the ACR.EULAR criteria as well as established SGUS scoring systems, particularly in terms of sensitivity. These findings suggest that MSOT may serve as a promising, non-invasive, addition to the current diagnostic workup for SjD. Its high sensitivity may facilitate earlier diagnosis and reduce the need for invasive diagnostic procedures, such as salivary gland biopsy.

Our results align with findings from MSOT studies in other (auto-)inflammatory diseases. Prior studies in Crohn’s disease and pediatric inflammatory bowel disease reported increased hemoglobin-related signals in inflamed intestinal tissues compared to healthy controls, consistent with increased perfusion and hyperemia associated with active inflammation [20], [41]. Similarly, patients with peripheral arterial disease (PAD) have shown significant differences in hemoglobin content between PAD patients and healthy subjects [42]. These studies underscore MSOT’s ability to characterize inflammatory activity based on vascular parameters.

A key advantage of MSOT is its integration with conventional ultrasound platforms, allowing seamless acquisition of both structural and functional information. In our cohort, MSOT demonstrated markedly higher sensitivity compared to SGUS scoring systems, while maintaining comparable specificity. Although literature reports SGUS sensitivity and specificity at 67 % and 94 %, respectively, whereas we observed a substantially lower SGUS sensitivity of 30 %, with specificity ranging from 85 % to 100 % [12]. This discrepancy may reflect differences in our study population, and therefore the added value of MSOT may be overestimated and should be interpreted with caution. Nonetheless, MSOT still outperformed the SGUS sensitivity as reported in literature, and thus could offer detection of functional changes, such as alterations in microvascularity and tissue oxygenation, that may precede the structural damage captured by SGUS. As these microvascular changes are characterized by slow-flowing blood, MSOT may have a diagnostic advantage over Color and Power Doppler ultrasound, which are less sensitive to low-velocity flow [43], [44]. Although Doppler ultrasound has shown increased blood flow towards salivary glands in SjD patients, its diagnostic accuracy is known to be highly dependent on operator experience [27], [45]. In contrast, MSOT has demonstrated consistent image quality and interpretability across users with varying levels of experience [46]. This suggests that MSOT may provide a more robust and operator independent tool for identifying microvascular changes in SjD, potentially enabling earlier and more reliable diagnosis.

While the results of this study are promising, several limitations should be acknowledged. The small cohort size limits the generalizability of our findings compared to previously published studies (e.g., in terms of SGUS sensitivity). Larger and more diverse patient populations will be necessary to validate the MSOT scoring system and confirm its diagnostic utility. Additionally, Color and Power Doppler ultrasound was not routinely collected, as they were not part of the standard imaging procedure during time of enrollment. Their inclusion may have supported the observed increases in total hemoglobin by detecting potential blood flow alterations, reinforcing the hypothesis of vascular changes. Although ROI placement was standardized in size and location (superficial gland area), operator variability still remains a concern. Development of objective criteria for ROI selection is necessary to reduce operator-dependent variability and improve measurement reproducibility. The use of smaller, standardized ROIs used in this study, intended to mitigate the effects of signal reduction at the edges and in deeper regions of the FOV, could provide a foundation for enhancing the precision and reproducibility of MSOT measurements [47].

Despite the promising results, MSOT still faces several technical limitations that must be addressed in order to establish broad clinical implementation. One major constraint is the limited frequency range of the ultrasound component; the 3.4 MHz transducer used in this study restricts spatial resolution compared to conventional ultrasound systems that operate across 2–15 MHz, depending on the required imaging depth [48]. While most MSOT platforms offer fixed frequencies, integrating a multi-frequency transducer could enhance versatility and image quality. Another challenge lies in improving fluence correction and artifact reduction, particularly for deep tissue imaging [49]. These improvements would contribute to the generation of high-quality, well annotated datasets that can support the development of tissue- or indication specific imaging configurations [50]. Moreover, advances in image reconstruction, especially trough deep learning methods, can significantly improve MSOT’s ability to produce anatomically accurate, high quality images for specific tissue types or imaging depths [51]. Together, these hardware and software optimizations, coupled with high-quality annotated datasets, will be essential to achieve clinically applicable MSOT imaging protocols.

Future research should focus on validating our findings in a larger and possibly more representative (i.e. with improved SGUS sensitivity) cohort. In a larger cohort, it would be of interest to evaluate the accuracy of the ACR-EULAR criteria when MSOT is incorporated, and to determine how the diagnostic performance of the criteria changes if each individual criterion is replaced by MSOT, similar to the approach used for SGUS [14]. Also, since our study was not specifically designed to assess patients at early disease stages, future studies that include early or preclinical SjD cohorts will be necessary to determine whether MSOT can indeed detect microvascular alterations before structural changes become apparent. As evidence accumulates, the development of a standardized, quantitative MSOT protocol is key to successful clinical implementation, including ROI definition, analysis tools, tissue- or indication specific imaging configurations, and imaging procedures. Beyond SjD classification, future studies should investigate its capability in differentiating SjD from other auto-immune diseases affecting the salivary glands, such as sarcoidosis, HIV, HepC and IgG4 related diseases [52]. The development of targeted MSOT tracers may be needed to establish disease specificity. For example, tracers targeting IL-2 or somatostatin receptors, both overexpressed in the salivary glands of SjD patients, could provide molecular-level specificity along with the functional information derived from intrinsic tissue chromophores such as HbR and HbO2 [53], [54].

## 5 Conclusion

To our knowledge, this is the first study to demonstrate that MSOT can serve as a non-invasive diagnostic tool for SjD. MSOT showed superior sensitivity compared to the diagnostic tests of the ACR-EULAR criteria and established SGUS scoring systems, while maintaining similar specificity as SGUS. Further research in needed to confirm these findings, address current technical limitations and determine whether MSOT can be easily integrated into standard diagnostic workup. MSOT holds promise to establish a more timely diagnosis of SjD and reduce reliance on invasive procedures by offering objective and non-invasive assessment of salivary gland involvement.

## CRediT authorship contribution statement

**Riemer H.J.A. Slart:** Writing – review & editing. **Alja Stel:** Writing – review & editing. **Max J.H. Witjes:** Writing – review & editing. **Konstantina Delli:** Writing – review & editing, Writing – original draft. **Andor W.J.M. Glaudemans:** Writing – review & editing. **Schelto Kruijff:** Writing – review & editing, Supervision. **Gooitzen M. van Dam:** Writing – review & editing. **Jasper Vonk:** Writing – review & editing, Writing – original draft, Supervision, Methodology, Conceptualization. **Rik de Jong:** Writing – review & editing, Writing – original draft, Methodology, Investigation, Formal analysis, Conceptualization. **Milou E. Noltes:** Writing – review & editing, Writing – original draft, Methodology, Conceptualization. **Hendrika Bootsma:** Writing – review & editing.

## Ethics approval

The study protocol was approved by the Medical Ethics Committee Groningen, and the trial was performed in accordance with the principles of Good Clinical Practice guidelines and the Declaration of Helsinki

## Ethics statement

The patients in this manuscript have given written informed consent.

## Funding

This research did not receive any specific grant from funding agencies in the public, commercial, or not-for-profit sectors.

## Competing interest

GMvD is founder, shareholder, and CEO of TRACER Europe BV (Groningen, the Netherlands). None of the other authors reported any potential conficts of interest.

## Declaration of Competing interest

The authors declare that they have no known competing financial interests or personal relationships that could have appeared to influence the work reported in this paper.

## Data Availability

Data will be made available on request.

## Footnotes

Appendix A Supplementary data associated with this article can be found in the online version at doi:10.1016/j.pacs.2025.100778.
