# Supplementary material for: Multispectral optoacoustic tomography of salivary glands in patients with clinically suspected Sjögren’s disease: A pilot study
Source: Photoacoustics. 2025 Nov 1;46:100778. doi: 10.1016/j.pacs.2025.100778 (PMC12629920; doi:10.1016/j.pacs.2025.100778)
Supplement: Supplementary file 1 — Supplementary material [file mmc1.docx]

**Supplementary Material**


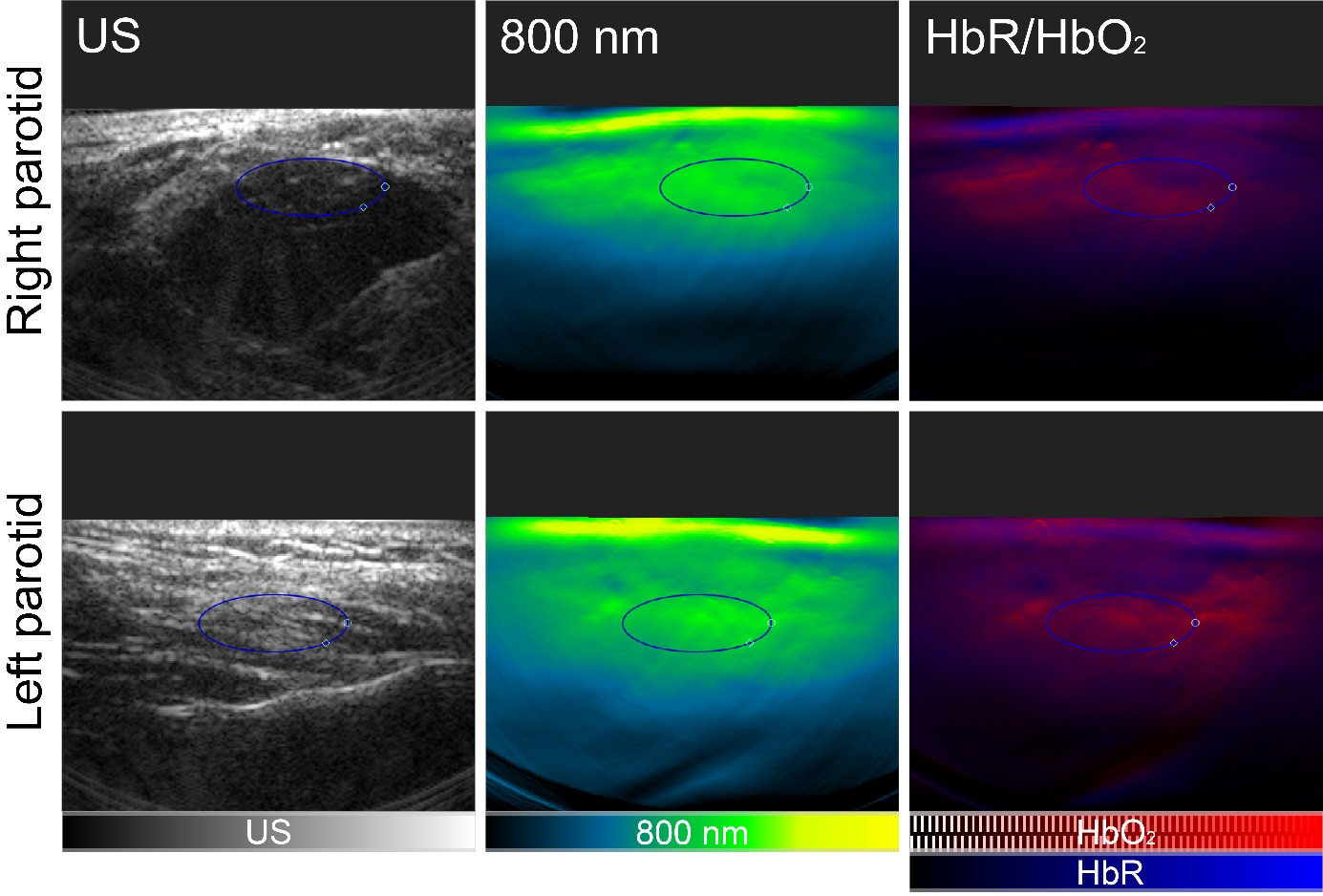


**Supplementary Fig. S1** Exemplary US (ultrasound), 800 nm SWL (single wavelength), and HbR (deoxyhemoglobin) /HbO_2_ (oxyhemoglobin) images of a MALT Lymphoma case (right parotid) an the left parotid gland of the same patient, representing a SjD (Sjögren’s disease) case.
