# Supplementary material for: Multispectral optoacoustic tomography of salivary glands in patients with clinically suspected Sjögren’s disease: A pilot study
Source: Photoacoustics. 2025 Nov 1;46:100778. doi: 10.1016/j.pacs.2025.100778 (PMC12629920; doi:10.1016/j.pacs.2025.100778)
Supplement: Supplementary file 2 — Supplementary material [file mmc2.docx]

**Supplementary Material**

**Supplementary table S1** MSOT parameters of patients classified as SjD and non-SjD based on ACR-EULAR criteria.

| Parotid gland | | | | | |
| --- | --- | --- | --- | --- | --- |
|  | SjD | | Non-SjD | | p-value |
|  | Median (a.u.) | IQR | Median (a.u.) | IQR |  |
| ROI depth, mm | 5.5 | 4.7 – 6.4 | 5.5 | 4.7 – 6.5 | 0.3662 |
| 700 nm | 427.9 | 389.4 – 460.9 | 377.5 | 330.5 – 413.9 | 0.0035** |
| 730 nm | 434.9 | 395.5 – 475.0 | 382.2 | 365.5 – 402.8 | 0.0038** |
| 760 nm | 428.7 | 400.0 – 463.1 | 374.3 | 361.6 – 399.1 | 0.0038** |
| **800 nm** | **403.1** | **374.6 – 428.0** | **344.9** | **335.4 – 366.6** | **0.0035**** |
| 850 nm | 389.9 | 360.4 – 424.3 | 334.5 | 323.3 – 359.2 | 0.0042** |
| HbR | 0.213 | 0.188 – 0.231 | 0.191 | 0.166 – 0.203 | 0.0047** |
| HbO_2_ | 0.247 | 0.225 – 0.283 | 0.218 | 0.120 – 0.245 | 0.0179** |
| **HbT** | **0.469** | **0.437 – 0.507** | **0.407** | **0.390 – 0.431** | **0.0028**** |
| Submandibular gland | | | | | |
| ROI depth, mm | 8.0 | 7.4 – 9.3 | 8.2 | 7.5 – 9.6 | 0.146 |
| 700 nm | 383.0 | 362.3 – 414.9 | 354.7 | 334.4 – 384.2 | 0.0439* |
| 730 nm | 422.4 | 359.7 – 432.7 | 371.5 | 356.4 – 395.5 | 0.0186* |
| 760 nm | 389.4 | 345.4 – 406.9 | 348.6 | 334.8 – 375.6 | 0.0219* |
| **800 nm** | **381.8** | **338.1 – 406.4** | **343.4** | **320.5 – 360.8** | **0.0171*** |
| 850 nm | 362.7 | 327.1 – 380.4 | 326.0 | 302.1 – 342.7 | 0.0186* |
| HbR | 0.184 | 0.175 – 0.200 | 0.176 | 0.167 – 0.185 | 0.0668 |
| HbO_2_ | 0.247 | 0.204 – 0.270 | 0.215 | 0.193 – 0.223 | 0.0763 |
| **HbT** | **0.445** | **0.390 – 0.461** | **0.392** | **0.370 – 0.416** | **0.0202*** |

Abbreviations: a.u., arbitrary unit; IQR, interquartile range; ROI, region of interest; HbR, deoxygenated hemoglobin; HbO_2,_ oxygenated hemoglobin; HbT, total hemoglobin; The single wavelength and unmixing spectra parameters with the greatest significant p-value are in bold; * = p < 0.05, ** = p <0.005.

**Supplementary table S2** Optimal predictive values for the parotid and submandibular gland to predict the ACR-EULAR classification outcome.

| Parotid gland | | | | | | | |
| --- | --- | --- | --- | --- | --- | --- | --- |
|  | Cut-off value (a.u.) | Sensitivity (%) | Specificity (%) | NPV (%) | PPV (%) | AUC | Accuracy (%) |
| 700 nm | 424.7 | 61.5 | 85.7 | 54.6 | 88.9 | 0.778 | 70.0 |
| 730 nm | 413.5 | 69.2 | 85.7 | 60.0 | 90.0 | 0.775 | 75.0 |
| 760 nm | 406.6 | 73.1 | 85.7 | 63.2 | 90.5 | 0.775 | 77.5 |
| **800 nm** | **371.6** | **80.8** | **85.7** | **70.6** | **91.3** | **0.778** | **82.5** |
| 850 nm | 360.9 | 76.9 | 85.7 | 66.7 | 90.9 | 0.772 | 80.0 |
| HbR | 0.208 | 65.4 | 85.7 | 57.1 | 89.5 | 0.769 | 72.5 |
| HbO_2_ | 0.235 | 65.4 | 71.4 | 52.6 | 81.0 | 0.728 | 67.5 |
| **HbT** | **0.434** | **80.8** | **85.7** | **70.6** | **91.3** | **0.783** | **82.5** |
| Submandibular gland | | | | | | | |
| 700 nm | 376.2 | 56.0 | 78.6 | 49.0 | 82.9 | 0.697 | 64.1 |
| 730 nm | 408.1 | 60.0 | 92.9 | 55.6 | 94.0 | 0.729 | 71.8 |
| 760 nm | 380.7 | 56.0 | 92.9 | 53.2 | 94.0 | 0.723 | 69.2 |
| **800 nm** | **374.2** | **60.0** | **92.9** | **55.6** | **94.0** | **0.731** | **71.8** |
| 850 nm | 360.6 | 52.0 | 92.9 | 51.0 | 93.1 | 0.729 | 66.7 |
| HbR | 0.181 | 56.0 | 78.6 | 49.0 | 82.9 | 0.680 | 64.1 |
| HbO_2_ | 0.247 | 52.0 | 92.9 | 51.0 | 93.1 | 0.674 | 66.7 |
| **HbT** | **0.414** | **72.0** | **78.6** | **60.2** | **86.2** | **0.726** | **74.4** |

Abbreviations: a.u., arbitrary unit; AUC, area under the curve; HbR, deoxygenated hemoglobin; HbO_2,_ oxygenated hemoglobin; HbT, total hemoglobin; The single wavelength and unmixing spectra parameters with the best predictive ability are in bold.

**Supplementary table S3** **Determining the optimal gland count exceeding the MSOT cut-off value for predicting ACR-EULAR classification outcome**. The outcome with the greatest predictive ability is highlighted in bold. PPV (positive predictive value); NPV (negative predictive value).

| Cut-off value of positive gland(s) | ≥ 1 | **≥ 2** | ≥ 3 | ≥ 4 |
| --- | --- | --- | --- | --- |
| Sensitivity (%) | 100.0 (13/13) | **92.3 (12/13)** | 53.9  (7/13) | 30.8  (4/13) |
| Specificity (%) | 57.1 (4/7) | **100.0 (7/7)** | 100.0  (7/7) | 100.0  (7/7) |
| NPV (%) | 100.0 | **85.7** | 58.3 | 46.6 |
| PPV (%) | 81.3 | **92.3** | 100.0 | 100.0 |
| Accuracy (%) | 85.0  (17/20) | **95.0 (19/20)** | 70.0  (14/20) | 55.0  (11/20) |

**Supplementary table S4** SGUS and the diagnostic tests of the ACR-EULAR criteria versus the MSOT outcome (92% sensitivity and 100% specificity) as reference standard. UWS (unstimulated whole saliva flow), OSS (ocular staining score).

|  | Sensitivity  (%) | Specificity  (%) | NPV  (%) | PPV  (%) | AUC | Accuracy (%) |
| --- | --- | --- | --- | --- | --- | --- |
| Imaging modalities vs MSOT outcome | | | | | | |
| OMERACT | 33.3 | 87.5 | 46.7 | 80.0 | 0.604 | 55.0 (11/20) |
| HOCEVAR | 33.3 | 100.0 | 50.0 | 100.0 | 0.667 | 60.0 (12/20) |
| Diagnostic tests of the ACR-EULAR classification criteria vs MSOT outcome | | | | | | |
| Parotid biopsy | 83.3 | 62.5 | 71.4 | 76.9 | 0.729 | 75.0 (15/20) |
| Anti-SSA/Ro | 75.0 | 100.0 | 72.7 | 100.0 | 0.875 | 85.0 (17/20) |
| UWS | 66.8 | 50.0 | 50.0 | 66.7 | 0.583 | 60.0 (12/20) |
| OSS | 0 | 87.5 | 36.8 | 0 | 0.563 | 35.0 (7/20) |
| Schirmer | 58.3 | 50.0 | 44.4 | 63.6 | 0.542 | 55.0 (11/20) |
